# News and Notes

**Published:** 1904-11

**Authors:** 


### NEWS AND NOTES.

A VIRULENT outbreak of smallpox is reported from North Adams, Mass.

IN July two hundred and thirty-six ambulances were shipped from Indianapolis to Japan.

TWO HUNDRED AND THIRTY-THREE cases of typhoid fever are reported from the District of Columbia.

DR. HARRIS G. MINTER and Miss Alma Reddoch were married at Jakin, Ga., October 19. Congratulations.

DR. P. A. LOVERING, surgeon U. S. N., has been ordered to the chair of tropical diseases at the Naval Medical School in Washington.

A LEAGUE against dust has been formed in Paris. Its object is to impress on the public mind the dangers caused by the diffusion of microbes in dust.

DR. KENN W. MILLICAN, who since April, 1898, has held the position of associate editor of the *New York Medical Journal*, has resigned to take charge of the *St. Louis Medical Review*.

The Medical College of Georgia, Augusta, opened for the seventy-fourth year, October 3, with the largest class ever matriculated. Dr. DeSaussure Ford, the dean, delivered the opening address.

DR. JOHN M. SPENCE, Waresboro, representative from Ware county, was stabbed and seriously wounded September 23, by a man whom he accused of having circulated false and slanderous reports about him.

---

MAJOR J. F. CASE, city engineer of Manila, has laid before the Secretary of War and Chief of the Bureau of Insular Affairs at Washington plans for the proposed water-supply and sewerage system of Manila.

---

DR. R. H. THIGPEN has resigned as head interne at the City Hospital, Augusta, and has been succeeded by Dr. J. M. Sigmen. Dr. J. B. Carter, Jr., has been advanced to junior interne and Dr. Asbury Stall has been made ambulance surgeon.

---

IT is stated that Drs. Ott and Hirsch, the two physicians in attendance on the Czarina during her recent confinement, received a special fee of 100,000 roubles in honor of the birth of an heir to the Russian throne. The ordinary fee is 25,000 roubles.

---

MAJ. OLIVER B. SIMMONS, in charge of the South Mountain Camp Sanatorium on the Pennsylvania State Forestry Reservation at Mont Alto, Franklin county, has reported, says an exchange, that since the sanatorium for consumptives was opened in June, 1903, about sixty-five patients have been received, and at present there are thirty there, most of whom will spend the winter in the open. Of those who were received for treatment fifty per cent. have returned to their homes apparently cured.

---

DR. GEORGE F. BUTLER has severed his connection with the Alma Springs Sanitarium, at Alma, Michigan, where for nearly five years he has been medical superintendent, and has returned to Chicago, where he will henceforth limit his practice strictly to internal medicine. He will fill the chairs of professor of therapeutics in the College of Physicians and Surgeons, and professor of medicine in the Dearborn Medical College; he has also been appointed as one of the attending physicians in the Samaritan Hospital. Dr. Butler will continue to edit and publish his magazine, "How to Live," and it is understood that he has under way another medical work for a Philadelphia medical-book publisher.

---

JAHUS contains an article by Dr. Alex Johannessen on infant mortality in Norway, in which it is shown that this country has the lowest of any civilized land, the proportion for the whole country being 95 per 1000. In the towns it is higher (130) than in the country (86), and it is higher for boys (106) than for girls (89). As is the case everywhere, it is higher for illegitimate children (150) than for legitimate (93). It rises in the extreme north beyond the polar circle to 150. A peculiarity of the Norwegian infant mortality, and the chief cause of its low rate, is the absence of the usual summer rise due to infantile diarrhea. From investigation of the records of several parishes during the past two centuries, Dr. Johannessen finds a great improvement in the nineteenth as compared with the eighteenth century, the respective mortalities being 101 and 105; the bad record of the eighteenth mainly due to deaths during the month of January.

---

AN exchange states that the Paymaster-General's Department of the regular army reports that since the abolishment of the army post exchange, or the canteen, as it is familiarly called, the savings of the soldiers deposited with the paymaster's office have shrunk nearly $2,000,000. This is not of course an absolute demonstration that the soldiers have lost their thrifty habits; but the army officers and the persons who are intimately connected with the army are of the opinion that the money which was formerly stored away now goes in wild debauches to the dens, joints, and vile resorts of every description which have grown up on the outskirts of all army barracks and camps in large numbers since the passage of the law which forbade the sale of beer and light wines in the camps under strict regulation and under the supervision of the army officers. The strongest kind of support for the canteen has come from total abstainers, physicians, and chaplains, who have no objects to serve but to make the discipline good, the soldier efficient, and the army cleanly and decent.

